# Machine learning-driven exploration of drug therapies for triple-negative breast cancer treatment

**DOI:** 10.3389/fmolb.2023.1215204

**Published:** 2023-08-04

**Authors:** Aman Chandra Kaushik, Zhongming Zhao

**Affiliations:** ^1^ Center for Precision Health, School of Biomedical Informatics, The University of Texas Health Science Center at Houston, Houston, TX, United States; ^2^ Human Genetics Center, School of Public Health, The University of Texas Health Science Center at Houston, Houston, TX, United States; ^3^ MD Anderson Cancer Center, UTHealth Graduate School of Biomedical Sciences, Houston, TX, United States

**Keywords:** drug sensitivity, pharmacogenomics, *SETD7*, *SRARP*, *YIPF5*

## Abstract

Breast cancer is the second leading cause of cancer death in women among all cancer types. It is highly heterogeneous in nature, which means that the tumors have different morphologies and there is heterogeneity even among people who have the same type of tumor. Several staging and classifying systems have been developed due to the variability of different types of breast cancer. Due to high heterogeneity, personalized treatment has become a new strategy. Out of all breast cancer subtypes, triple-negative breast cancer (TNBC) comprises ∼10%–15%. TNBC refers to the subtype of breast cancer where cells do not express estrogen receptors, progesterone receptors, or human epidermal growth factor receptors (ERs, PRs, and HERs). Tumors in TNBC have a diverse set of genetic markers and prognostic indicators. We scanned the Cancer Cell Line Encyclopedia (CCLE) and Genomics of Drug Sensitivity in Cancer (GDSC) databases for potential drugs using human breast cancer cell lines and drug sensitivity data. Three different machine-learning approaches were used to evaluate the prediction of six effective drugs against the TNBC cell lines. The top biomarkers were then shortlisted on the basis of their involvement in breast cancer and further subjected to testing for radion resistance using data from the Cleveland database. It was observed that Panobinostat, PLX4720, Lapatinib, Nilotinib, Selumetinib, and Tanespimycin were six effective drugs against the TNBC cell lines. We could identify potential derivates that may be used against approved drugs. Only one biomarker (*SETD7*) was sensitive to all six drugs on the shortlist, while two others (*SRARP* and *YIPF5*) were sensitive to both radiation and drugs. Furthermore, we did not find any radioresistance markers for the TNBC. The proposed biomarkers and drug sensitivity analysis will provide potential candidates for future clinical investigation.

## Introduction

Triple-negative breast cancer (TNBC) is an aggressive tumor that accounts for ∼10%–15% of all breast cancer (BC) subtypes and has a bad prognosis (Haffty et al., 2006; Dent et al., 2007; Mittendorf et al., 2014; Sabatier et al., 2015; Luen et al., 2016; Denkert et al., 2018). Pharmacogenomics predictions deal with genomic changes in our body due to response to medications. It is a growing field of study that includes the development of drugs, repurposing, selection of patients for clinical studies, and recommendations for individualized therapy. It demonstrates how the body processes and metabolizes various medications (Barretina et al., 2012; Seashore-Ludlow et al., 2015; Haverty et al., 2016; Iorio et al., 2016). To build predictive models, we can use pharmacological response data and molecular data from a variety of cell lines from these databases. However, there are restrictions in determining medication response (Papillon-Cavanagh et al., 2013; Jang et al., 2014). Noise in the data along with the presence of more characteristics than sample size (i.e., predictors/variables), insufficient characterization of the omics data, and the lack of dynamic nature of molecular data are the overall factors that render drug response prediction more difficult (Kalamara et al., 2018). Another significant issue is that we can not rely on the pharmacogenomic correlations generated from drug response. Several studies have found that inconsistencies in utilized experimental techniques and processing of data resulted in reported inconsistency (Haibe-Kains et al., 2013; Consortium CCLEConsortium GoDSiC, 2015; Bouhaddou et al., 2016; Geeleher et al., 2016; Safikhani et al., 2016; Mehmood et al., 2023).

In simpler terms, drug sensitivity can be defined as the amount of activity achieved on a target (in this case, cell lines). It is measured using various methods including area above the curve (AAC) and IC50 values, whereas drug resistance refers to the resistance of a target to a drug or specific compounds, which may be triggered by mutations or overdosing. This is correlated to biomarkers, which are entities that play a critical role in tumor survival. Radiotherapy is the most effective cancer treatment (Baumann et al., 2016). For quick and efficient results, along with chemotherapy, radiation is also considered. Radiation’s physical accuracy has been improved by recent technological advances, yielding higher cure rates and lowering toxicity (Baumann et al., 2016).

Numerous methods and approaches have been developed to solve the drug response prediction problem, including normalized regression techniques (i.e., least absolute shrinkage and selection operator (LASSO), elastic net, and ridge regression) (Geeleher et al., 2014; Falgreen et al., 2015; Fang et al., 2015; Aben et al., 2016), support vector machines (SVM) (Dong et al., 2015), random forest, neural networks, deep learning (Menden et al., 2013; Ding et al., 2018), and logical models (Ammad-Ud-Din et al., 2014; Ammad-Ud-Din et al., 2016). [Ali and Aittokallio (2019)] provide a review with wider detail. There has yet to be reported a comprehensive study of procedures of model training which is based on data from large cell line screenings as well as radiation data. In this study, we aimed to fill these gaps, in turn improving the accuracy of drug response prediction and discovering new biomarkers for sensitivity pertaining to drugs as well as radiation. Predictive performance was assessed by tenfold cross-validation and sampling of five models trained by various machine learning methods, i.e., elastic net (Heiss et al., 2021), LASSO (Huang et al., 2020), ridge (Arashi et al., 2021), random forest (Schonlau and Zou, 2020), and support vector machine (Pisner and Schnyer, 2020).

First, the sensitivity data for the drugs and cell lines data were retrieved from the two datasets [Cancer Cell Line Encyclopedia (CCLE) and Genomics of Drug Sensitivity in Cancer (GDSC)] and drug activity was examined using the IC50 and the area above the curve (AAC) graphs by us. This was done for the 16 shortlisted drugs considered in this study. Then we used the multivariate machine learning models to predict the accuracy of drug sensitivity on the cancer cell lines and shortlisted the best-performing drugs. We manually searched for potential biomarkers after shortlisting biomarkers from molecular data. Furthermore, for the radiation sensitivity data, the Cleveland database was used because we wanted to find biomarker signatures that were sensitive to both chemotherapy and radiation. Identifying signatures associated with radiosensitivity or radioresistance was possible with the RadioGx package.

## Materials and methods

**FIGURE 1:**
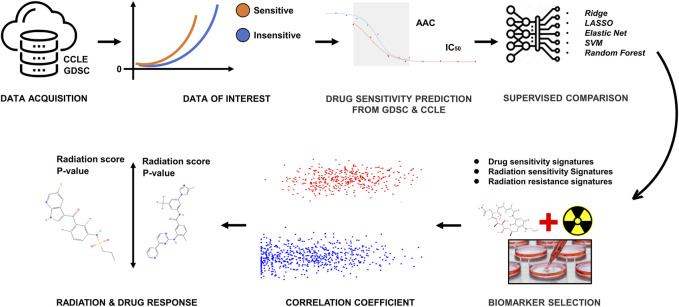
The methodological pipeline of the current study.

We curated a collection of cancer cell line screens from two different data sources for this study: Genomics of Drug Sensitivity in Cancer (GDSC) and Cancer Cell Line Encyclopedia (CCLE) (Yang et al., 2012; Wang et al., 2022). Each dataset contains a panel of cancer cell lines that have been drug-tested, and CCLE includes detailed genetic characterization of a large panel of human cancer cell lines. GDSC also contains information on the drug sensitivity of cancer cells and the molecular markers of drug response. Both datasets have some overlap in both cells and drugs. Our literature survey (Chavez et al., 2010; Gupta et al., 2016) defined 16 drugs and 10 cell lines on the basis of FDA approval to use for this study. To incorporate information on radiation sensitivity in this study, we also used the Cleveland1.0 (Boeckman et al., 2005) database. The overall workflow is given in Figure 1.

### Examining and extracting data of interest

We used two packages, PharmacoGx (Mahmoud and Haibe-Kains, 2020) and RadioGx (Trendowski et al., 2021), to analyze the datasets and extract the drug sensitivity data for each cell line. PharmacoGx package has been used for drug sensitivity while RadioGX has been used for radiosensitivity and radioresistance. All of the information in PharmacoGx is provided as R objects, containing both pharmacological and molecular information from each study for analysis. The RadioGx program provides a standardized data format for storing the results of radiogenomic experiments. The relationship between various cancer cell lines is investigated, as well as their response to various dosages and types of ionizing radiation. The object structure of both packages is strikingly similar. So, a joint interface is available for accessing the considerable data contained in these objects. The PSet and RSet store three types of data in general: metadata/annotations, molecular data, and treatment response data.

### Modeling the sensitivity data

The drug dose-response curve function can be used to plot drug dose-response data from PharmacoSet objects (Ma et al., 2020). In each dataset, it helps in plotting the drug dose-response curves for the combination of drugs against specific cell lines which further allows direct comparisons of data between the two datasets when a list of PharmacoSets, a name for drug against cell line is given.

### Drug sensitivity prediction

In pharmacogenomic studies, cancer cell lines were also tested for their dose-dependent response by increasing concentrations of various compounds from which IC50 values (Thorarensen et al., 2021), the area above the curve (AAC) (Govindaraj et al., 2020), and viability at 1 µM were computed. The IC50 of an inhibitor is the specific concentration for which the response is one-half the original response. The AAC is a more robust metric that is normalized against dose range and is defined as the area above the dose-response curve for the tested drug concentrations.

### Consistency of CCLE and GDSC datasets

We examined the consistency of the GDSC and CCLE databases and discovered that the names of the cells and drugs utilized in the datasets were not identical. However, we used PharmacoGx to overcome these disparities and conducted a comparison analysis between the two datasets. The hgu133a platform was used to profile GDSC, whereas the more comprehensive hgu133plus2 platform was used to profile CCLE. While the hgu133a platform is essentially a subset of the hgu133plus2 platform in this case, Ensemble Gene IDs summarise the expression information in PharmacoSet objects, making it possible to compare datasets from different platforms.

The downloadPSet utility was used to import the datasets from storage for testing consistency between them. To obtain the common intersect among the datasets, the intersectPSet was used. We created a breakdown of the gene expression and drug sensitivity metrics for both datasets, leaving one gene expression pattern and one sensitivity profile per cell line within each dataset. The Pearson correlation coefficient was then used to compare the gene expression and susceptibility metric between the datasets.

### Reliability assessment via robust concordance index (rCI)

We used the robust Concordance Index (rCI) to examine the concordance of multiple pharmacogenomic data sets (rCI) (Salisu et al., 2020; Smirnov et al., 2021). The robust concordance index (rCI) is used in cell line-based drug screening to estimate the probability that two randomly-chosen cell lines are ranked identically and that there is no repetition across biological replicates based on their response to drugs. We observed that noise in drug screening can be taken into account, and that responsive ranking of cell lines with similar AAC values may contain errors; however, the rCI only calculates cell line pairings with a drug sensitivity discrepancy greater than the threshold value.

### Machine learning-based validation

Ridge, elastic net, RF, LASSO, and SVM are some of the commonly used multivariate machine learning algorithms employed in our study. Multiple metrics have been used to evaluate the performance, including the Pearson correlation coefficient, concordance index, and robust concordance index.

#### Ridge method

L2 regularization is used in ridge regression, which introduces the given penalty element to the OLS equation.

$$+\lambda \sum_{j=0}^{p}w_{j}^{0}$$ (i)

The L2 term is directly proportional to the square of the coefficients’ magnitude. If lambda (λ) is 0, the formula is the basic OLS, but if it is bigger than zero, a restriction is added to the coefficients. This restriction leads to reduced coefficients (also known as shrinkage), which tend to zero as lambda increases. Reduced coefficients result in a reduced variance and, as a result, a smaller error value. As a result, while ridge regression reduces the intricacy of a model, it does not lower the number of variables; rather, it reduces the influence of those variables.

#### LASSO method

The L1 penalty term is used in lasso regression, which refers to the least absolute shrinkage and selection operator. The penalty for L2 is equivalent to the absolute amount of the coefficients’ magnitude:

$$+\lambda \sum_{j=0}^{p}\left|w_{j}\right|$$ (ii)

A lambda value of zero, like ridge regression, spits out the fundamental OLS equation; however, with the right lambda value, lasso regression can push several coefficients to zero. The greater lambda is, the more characteristics are reduced to zero. This can completely exclude some characteristics and leave us with a selection of predictors that can assist reduce the multi-collinearity and complexity of the model. Predictors that haven't shrunk to zero are crucial, therefore L1 regularization permits for selecting features (sparse selection).

#### Elastic net method

The elastic net is a third regularly used regression model that contains penalties both from L1 and L2 regularization:

$$\frac{\sum_{i=1}^{n}{\left(y_{i}-x_{i}^{J}\hat{ß}\right)}^{2}}{2n}+\lambda \left(\frac{1-\alpha }{2}\sum_{J=1}^{m}\hat{{ß}_{j}^{2}}+\alpha \left.\sum_{J=1}^{m}|\right|\hat{{ß}_{j}|}\right)$$ (iii)

Elastic net allows us to tweak the alpha parameter in addition to specifying and picking a lambda value, where 𝞪 = 0 refers to the ridge and 𝞪 = 1 to lasso. Simply expressed, if alpha is set to 0, the penalty function reduces to an L1 (ridge) term, and if alpha is set to 1, the penalty function lowers to the L2 (lasso) term. As a result, we may improve the elastic net by selecting an alpha value between zero and 1. For sparse selection, this effectively shrinks certain coefficients and sets others to 0. Finally, in each category, SVM and random forests are effective strategies (Sirsat et al., 2020).

#### Random forest algorithm

The RF method is an ensemble approach that employs a large number of classification and regression trees (CART) (Breiman et al., 2017). The bootstrapped samples and aggregated model outputs are used to train these trees. Bagging avoids the models from overfitting and ensures that it generalizes effectively. Each tree adjusts the judgment of its child nodes to maximize the quantity of freshly obtained information as it grows. The Gini impurity, which is the same as the Gini index, may be used to express it and is computed as follows:

$$Gini\;Impurity=1-\sum pj\left(1-pj\right)$$ (iv)

where pj is the probability of an element being categorized into a specific class (Sarica et al., 2017). Each tree develops in such a way that the Gini impurity is minimized. Each tree is given a dataset that is jumbled at random and grows uniquely. These trees yield real-world effects, and the voted-for class is mostly chosen.

#### Support vector machine

The support vector machine technique can be used for both linear and non-linear data for classification as well as regression problems. Each data point is first projected onto an n-dimensional subspace, with n being the variety of attributes. The hyperplane that divides the data into two groups is then found, with the minimal proximity for both categories maximized and categorization mistakes reduced (Joachims, 1999).

We considered the GDSC data as input which contains RNA data. There was no need to split the data into training and testing because we wanted to use different databases for training and testing. The intersect function was used to interest the GDSC and CCLE data. To efficiently select potential features, we used the RStudio package maximum relevance minimum redundancy (mRMR) (Radovic et al., 2017). Keeping the 5-fold cross-validation, sampling (10), and features at 100 as the threshold, we ran the models using the dplyr (Silge and Robinson, 2016), caret (Kuhn, 2015), and randomForest (RColorBrewer and Liaw, 2018) packages in RStudio (Allaire, 2012). The performance was assessed using the Pearson correlation coefficient and concordance index.

### Similarity search using machine learning

Data retrieval for shortlisted targets, pre-processing of the bioactivity compounds, labeling of active compounds with the rule of five (Lipinski), descriptors calculation, and clustering of the molecules based on their fingerprint similarity were done for the three shortlisted compounds (detailed data presented in Supplementary Information).

1. Similarities search for Tanespimycin: First, we did data preparation or data labeling where we added a column for activity with a pIC50 of >= 6.0, and we found the number of active compounds was 210 while the number of inactive compounds was 198. Molecule encoding was done using the MACCS Method and we applied three classical machine learning approaches to classify our molecules namely, SVM, RF, and ANN, and the performance of the models where we fitted classical machine learning models on a train-test split of the data was observed. Splitting the data was reused for the two other classical models. We used test (x) and train (x) for the respective fingerprint splitting and test (y) and train (y) for the respective label splits, where the training data size was 326 and the test data size was 82.
2. Similarities search for Selumetinib: First, we did data preparation or data labeling where we added a column for activity with a pIC50 of >= 6.0, and we found the number of active compounds was 93 while the number of inactive compounds was 51. Molecule encoding was done using the MACCS Method and we applied three classical machine learning approaches to classify our molecules namely, SVM, RF, and ANN, and the performance of the models where we fit classical machine learning models on a train-test split of the data was observed. Splitting the data was reused for the two other classical models. We used test (x) and train (x) for the respective fingerprint splitting and test (y) and train (y) for the respective label splits, where the training data size was 115 and the test data size was 29.
3. Similarities search for Lapatinib: First, we did data preparation or data labeling where we added a column for activity with a pIC50 of >= 6.0, and we found the number of active compounds was 735 while the number of inactive compounds was 542. Molecule encoding was done using the MACCS Method and we applied three classical machine learning approaches to classify our molecules namely, SVM, RF, and ANN, and the performance of models where we fit classical machine learning models on a train-test split of the data was observed. Splitting the data was reused for the two other classical models. We used test (x) and train (x) for the respective fingerprint splitting and test (y) and train (y) for the respective label splits, where the training data size was 1,021 and the test data size was 256.

### Biomarker discovery

#### Drug sensitivity signatures

To search for drug sensitivity biomarkers, we obtained RNA molecular profiling data from the GDSC and CCLE databases. The PharmacoGx package’s functions were used for the generation of signatures of molecular features which correlate with individual reactions to particular compounds.

#### Radiation sensitivity signatures

The ability to determine gene signatures for a cell line from a radiosensitivity experiment is what makes the RadioGx package truly useful (Cleveland database). Cell lines of interest can be chosen by any researcher, and a molecular signature that correlates to specific molecular features along with a given sensitivity profile can be computed. The identification of signatures associated with radiosensitivity or radioresistance can be performed thereafter.

##### Associating sensitivity signatures between radiation and drug response

For *in vitro* model systems, RadioGx allows one to compute the correlation signatures of molecular features with the response to treatment A natural question is how the signature for gamma radiation will compare to the signatures for the six shortlisted drugs on three biomarkers. This can be used to generate hypotheses for combination therapies or to learn more about the mechanism of drugs in the body.

#### Drug-biomarker association

The association between molecular features and response to a given drug is modeled using a linear regression model adjusted for tissue source:

Y=β0+βiGi+βtT+βbBY=β0+βiGi+βtT+βbB (v)

Where YY stands for the drug sensitivity variable; GiGi , TT , and BB denote the expression of the gene, tissue source, and the experimental batch respectively and the regression coefficients are shown by and ββs . Aside from the fact that there is a link between drug sensitivity and tissue source, we measure the intensity of the gene-drug interaction by βiβi . The variables YY and GG are adjusted to compute the standardized coefficient (standard deviation = 1). To compute the validity and evaluate the significance of the gene-drug interaction, βiβi (two-sided *t*-test) is used. The false discovery rate (FDR) technique is then used to fix *p*-values for multiple testing. With biomarker discoveries across pharmacogenomic research from CCLE and GDSC data, we can predict the significance of the link between medications and associated reported biomarkers.

## Results

### Cell lines datasets

We have considered two types of cell lines data which include the drug sensitivity and the radiation sensitivity from the respective databases. RNA, RNA-Seq, copy number variation (CNV), mutational, and drug response data are examples of this data type. The details of the datasets are given in Table 1, while the triple-negative breast cancer cell lines taken from the literature survey (Chavez et al., 2010; Gupta et al., 2016) are given in Table 2.

**TABLE 1:** Data collected from multiple platforms. In case of radiation data, only gamma radiation is used.

Dataset	Data type	Platform	Samples
GDSC [GDSC_2020 (v2-8.2)]	RNA, RNA-Seq, CNV, Mutation, Drug response	IC_50_	1,084 cell lines × 215,780 drugs sensitivity
CCLE (CCLE_2015)	RNA, RNA-Seq, CNV, Mutation, Drug response	IC_50_	1,094 cell lines × 11,670 drugs sensitivity
Cleveland (2017)	RNA, RNA-Seq, CNV, Mutation	γ	540 cell lines × 1 radiation

**TABLE 2:** Human triple negative breast cancer cell lines features and drugs detail.

TNBC cell lines	Origin	Pathology	Grade	Age	Ethnicity	Molecular classification	P53	BRCA1	PI3KA pathway	Other features	Drugs
CAL-85-1	PE	AC	NA	45	NA	Basal B	NA	Wt	PTEN protein not expression	Cells described as luminal in some reports but this classification is not based on global gene expression	Panobinostat, PD-0325901, Selumetinib, Tanespimycin
BT-549	PE	IDC	NA	72	Caucasian	Basal B	Mut	Wt	PTEN homo deletion	NA	Lapatinib, Nilotinib, Paclitaxel, Panobinostat, PLX4720, Saracatinib, Sorafenib, Tanespimycin
BT-20	PE	IDC	NA	74	Caucasian	Basal A	Mut	WT	PI3CA mutation	Amplified EGFR	Panobinostat, Tanespimycin
HCC1187	PE	IDC	3	41	Caucasian	Basal A	Mut	WT	NA	Paired normal cell line derived from blood leukocytes established	Paclitaxel, Panobinostat
HCC1395	PE	IDC	3	43	Caucasian	Basal B	Mut	Homo Mut	NA	Paired normal cell lines derived from blood leukocytes and breast stroma established	Panobinostat, PD-0325901, Tanespimycin
HCC1806	PE	ASq	2	60	Black	NA	Mut	Wt	NA	Paired normal cell line derived from blood leukocytes established	Nilotinib, Panobinostat, Tanespimycin
MDA-MB-157	PE	IMC	NA	44	Black	Basal B	Mut	Wt	Wt		Panobinostat
MDA-MB-435	PE	IDC	NA	31	Caucasian	Basal B	Mut	Wt	Wt	The original cell line was derived from a patient with breast cancer but recent array based analysis have questioned whether some of the current clones have been contaminated with a melanoma cell line	Crizotinib, Nilotinib, Paclitaxel, Palbociclib, Panobinostat, PD-0325901, PLX4720, Selumetinib, Sorafenib, Tanespimycin
MDA-MB-436	PE	IDC	NA	43	Caucasian	Basal B	Mut	Homo Mut	Wt		Panobinostat, Tanespimycin
MDA-MB-468	PE	AC	NA	51	Black	Basal A	Mut	Wt	PTEN homo deletion	Amplified EGFR	Panobinostat, Tanespimycin

### Pharmacological profiles and drug-dose response

**FIGURE 2:**
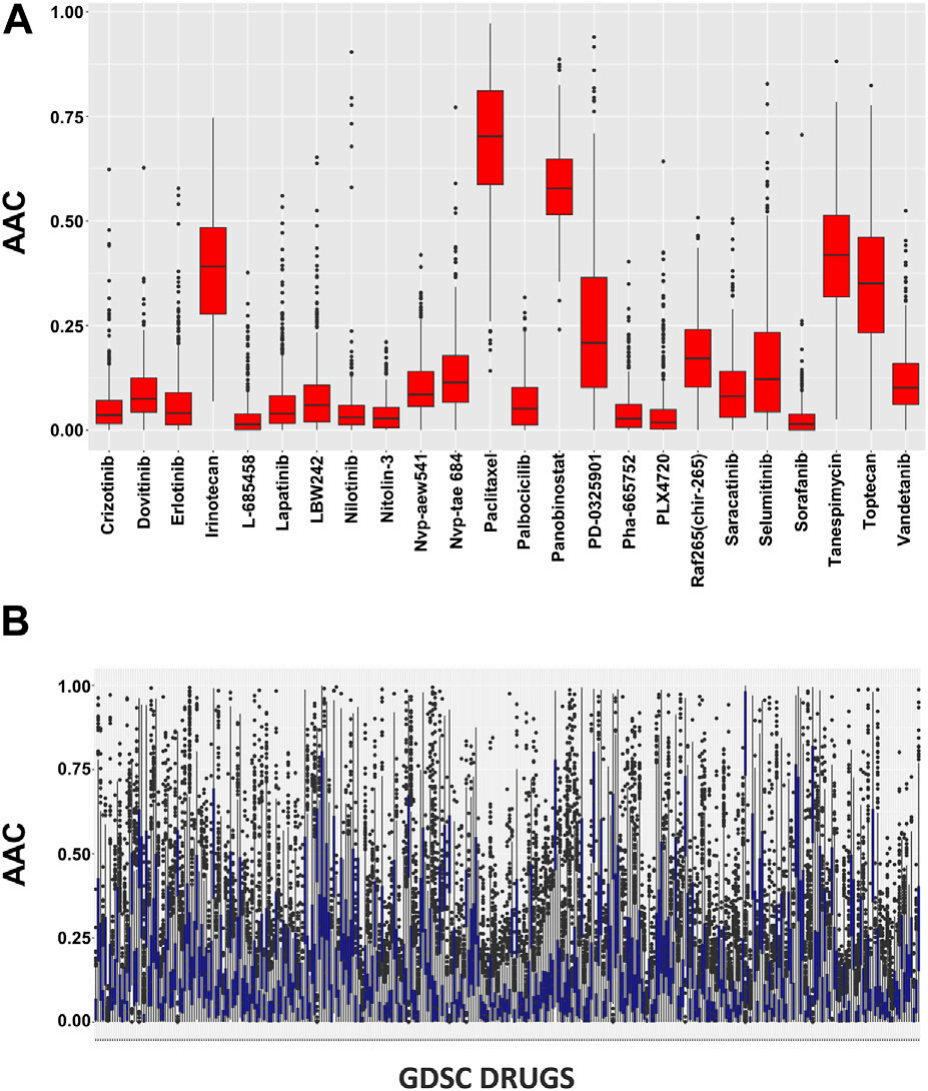
**(A)** Pharmacological profiles using the SummarizeSensitivityProfiles function to investigate the distributions of AAC values within CCLE dataset and **(B)** Pharmacological profiles using the SummarizeSensitivityProfiles function to investigate the distributions of AAC values within GDSC dataset.

In pharmacogenomic studies, cells were also evaluated for their reaction to increasing doses of various substances in pharmacogenomic research, and the minimum inhibitory concentration and AAC were calculated as a result (Figure 2). These pharmacological assessments are accessible using the PharmacoGx for all PSets.

To plot the drug-dose analysis results included in PharmacoSet objects, the drug dose response curve function was used. The AAC curves for all of the shortlisted drugs on 10 cell lines are included in the supporting documentation (Supplementary Figure S1).

The AAC calculation of the chosen drugs is summarized in Supplementary Figure S2 where all the cell lines show significant sensitivity towards the drugs. There is a clear difference between the CCLE and GDSC drug concentrations as CCLE has a maximum concentration of 10 uM while GDSC is restricted to 1 uM in 98% of the cases. The CCLE and GDSC curves show approximately 100% viability on a drug concentration of only 0.01 uM which proves its sensitivity towards the drugs. Among the shortlisted drugs, we observed that Panobinostat showed significant AAC values ranging from 0.37 to 0.64. Tanespimycin also had significant performance, ranging from 0.19 to 0.51, except for one cell line which shows a lower value of 0.6. The rest of the drugs had standard sensitivity for all the cell lines except Nilotinib which has the least performance ranging from 0.1 to 0.09.

Next, we calculated the IC50 of the above drugs based on the available data to examine how well drugs can inhibit the cancer cell lines **(** Supplementary Figure S3). For an IC50 summary of the drugs and cancer cell lines, refer to Supplementary Figure S3. We could not plot the IC50 for all six drugs because some of these drugs have not been tested experimentally and thus no data is available for them.

Here we only calculated the IC50 for Nilotinib, PLX4720, Sulemetinib, and Tanespimycin. These drugs demonstrated promising inhibitory activity against cancer cell lines with values ranging from 27 to 477 nM. Among them, Tanespimycin showed the best IC50 values which is consistent with the AAC performance. In the AAC evaluation, we marked Panobinostat and Tanespimycin and here we again chose Tanespimycin due to good inhibitory performance while no data is available for Panobinostat to compare the performance of both the drugs in terms of IC50 values.

**FIGURE 3:**
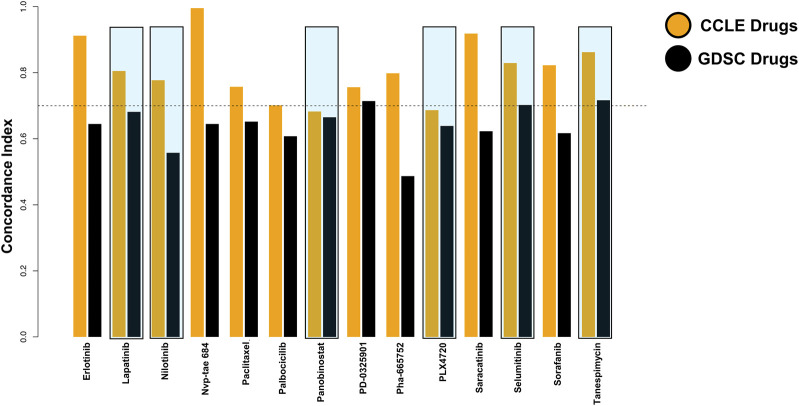
Consistency assessment between the two databases was improved by the concordance index.

Since the data were taken from two databases, we calculated the concordance index to examine the predictions made by the algorithm. The six drugs shortlisted by us were consistent among both databases (Figure 3). Within them, Tanespimycin showed a high concordance index among the top six drugs.

### Machine learning-based validation

A huge amount of drug sensitivity and drug compound data are available for cancer cell lines as a result of screening technologies. Computational techniques to analyze such data benefit anticancer therapeutics through the identification of molecular genomics determinants of drug sensitivity and the development of novel drugs for oncological targets. For drug sensitivity prediction, we used five machine-learning approaches: ridge, LASSO, elastic net, RF, and SVM. The GDSC database was used for training, while the CCLE database was used for testing.

**FIGURE 4:**
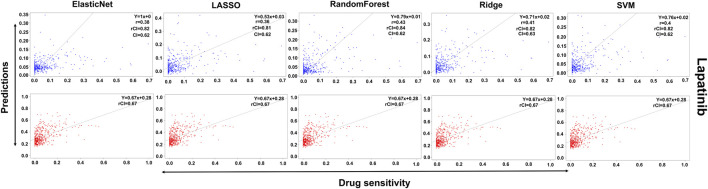
Predictions and validations using the five machine learning methods for Lapatinib. The blue dots represent predictions while red refers to the validations.

**FIGURE 5:**
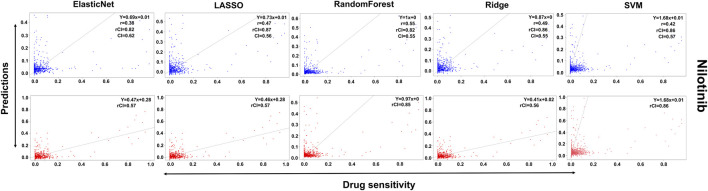
Predictions and validations using the five machine learning methods for Nilotinib. The blue dots represent predictions while red refers to the validations.

**FIGURE 6:**
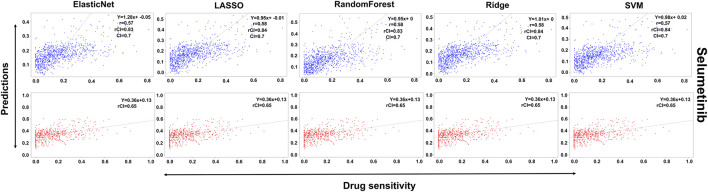
Predictions and validations using the five machine learning methods for Selumetinib. The blue dots represent predictions while red refers to the validations.

**FIGURE 7:**
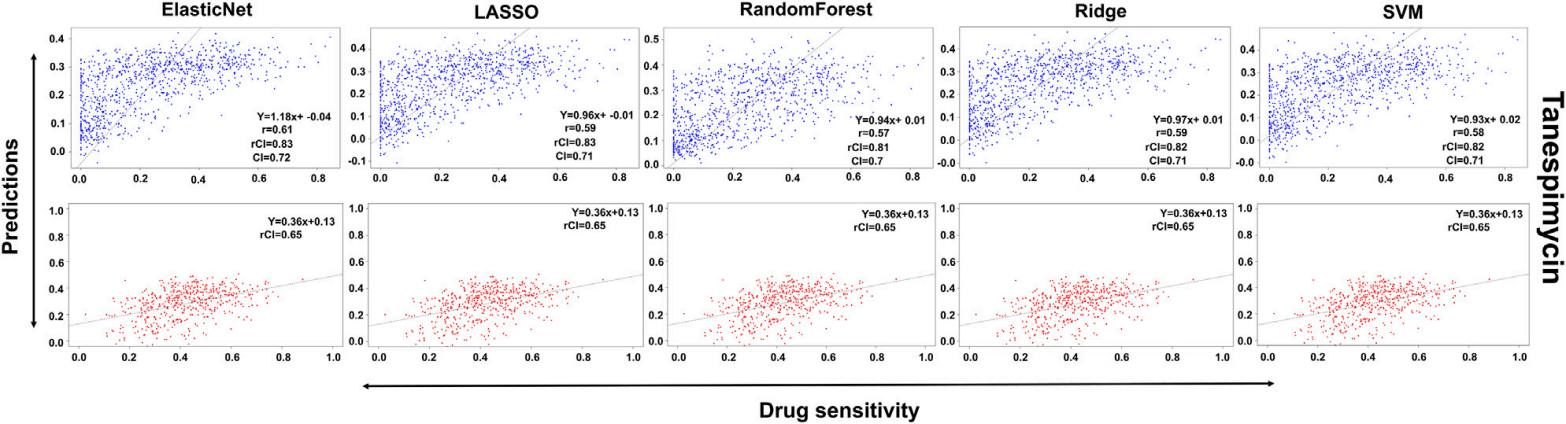
Predictions and validations using the five machine learning methods for Tanespimycin. The blue dots represent predictions while red refers to the validations.

In the case of Lapatinib (Figure 4), we observed that the elastic net, SVM, and ridge methods had an accuracy of 82%, which is better than LASSO (81%) but lower than RF which had the highest accuracy of 84%. In the case of Nilotinib (Figure 5), the least accuracy was observed for elastic net (80%) while the LASSO gains the highest accuracy of 87%. The RF was 82% accurate while the SVM and Ridge both have an accuracy of 86%. For the drug Panobinostat, (Supplementary Figure S4), the three models (ridge, LASSO, and SVM) showed a higher accuracy of 78% while RF and ElasticNet had an accuracy of 76% and 77% respectively. The highest accuracy in the case of PLX-4720 (Supplementary Figure S5) was observed using the RF which equaled 87%. Ridge was 86% accurate while LASSO, Elastic Net, and SVM had an accuracy of 86%, 83%, and 86% respectively. Similarly, Selumetinib also gained a higher accuracy from three methods (ridge, LASSO, and SVM) while elastic net and RF had an accuracy of 83% (Figure 6). One of the top drugs Tanespimycin (Figure 7) had the highest accuracy on two different methods (LASSO and elastic net) which equaled 83%. The lowest accuracy was observed in the case of RF (81%) while ridge and SVM had accuracies of 82%. As the validation was performed on CCLE data, a clear difference in validation accuracies was observed.

In contrast to the remaining compounds, Lapatinib had a validation accuracy of 67% across all five methods. Just like Lapatinib, 67% accuracy was observed for Panobinostat on all five models. The accuracy in the case of elastic net and LASSO was 57%, ridge performed the least with an accuracy of 56%, RF gained an accuracy of 85% while SVM had a higher accuracy of 86% for Nilotinib. All the models gave the same accuracy of 57% and 64% for PLX47-20 and Sulumetinib. Tanespimycin had an accuracy of 65%.

To summarize, only Nilotinib had different validation accuracies on different models while all the other drugs had the same validation accuracy on all five models. But Nilotinib was also observed to have a higher accuracy of 86%.

### Similarity search using machine learning

**FIGURE 8:**
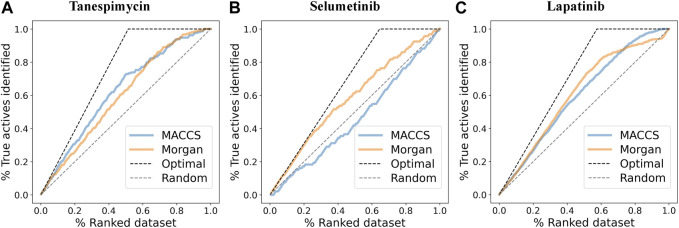
**(A)** Enrichment plots where the pIC50 (log *p*-value) cutoff was used to discriminate between active and inactive molecules (cutoff was 6.3) and find the enrichment for MACCS and Morgan fingerprints for Tanespimycin. **(B)** Enrichment plots to discriminate between active and inactive molecules for Selumetinib. **(C)** Enrichment plots to discriminate between active and inactive molecules for Lapatinib.

Data retrieval for shortlisted targets, pre-processing and labeling of the bioactivity compounds, fingerprint descriptors calculation, and clustering of the molecules based on their fingerprint similarity were done for all three shortlisted molecules (detailed data presented in Figure 8 and Supplementary Information).

**FIGURE 9:**
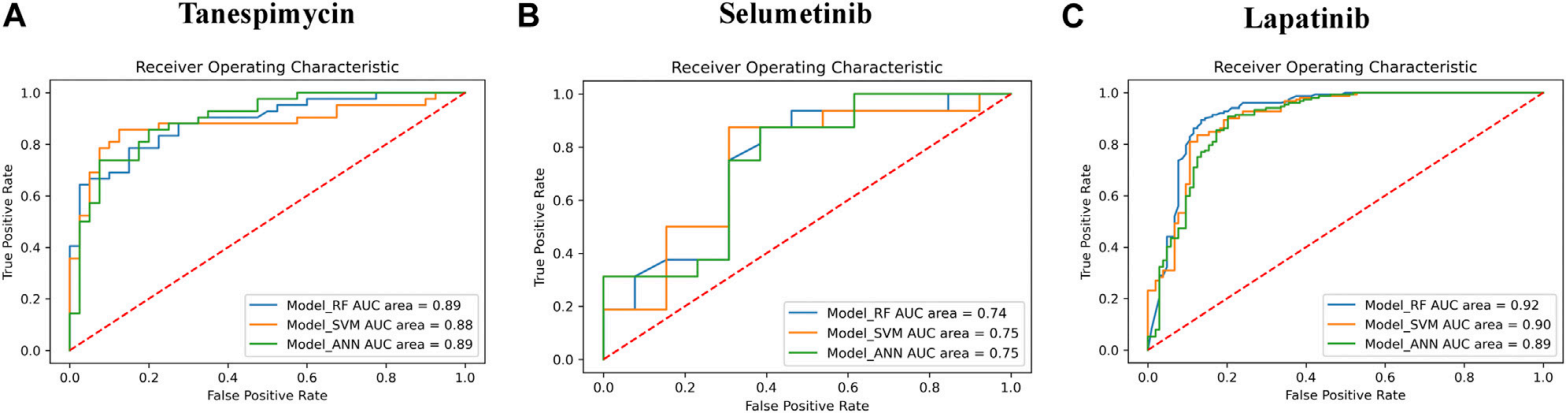
**(A)** The performance of three different models (RF, SVM, and ANN) for Tanespimycin. **(B)** The performance of three different models (RF, SVM, and ANN) for Selumetinib. **(C)** The performance of three different models (RF, SVM, and ANN) for Lapatinib.

1. Similarities search for Tanespimycin: The random forest classifier was applied where the set model parameter for random forest estimators was 100, and the number of trees to grow criterion (entropy) and number cost function were optimized for a split. We observed that the sensitivity for RF was 0.79, the specificity for RF was 0.82, and the AUC for RF was 0.89. The support vector classifier was applied where the set model parameters for the SVM kernel were rbf, C value of 1, gamma value of 0.1, and the probability was True. We observed that the sensitivity for SVM was 0.79, the specificity was 0.90, and the AUC for SVM was 0.88. A neural network classifier was applied where the set model parameters for ANN hidden layer sizes were 5 and 3, and the random state was seed We observed that the sensitivity for ANN was 0.74, the specificity was 0.82, and the AUC for ANN was 0.89, as shown in Figure 9A; Table 3. We performed cross-validation experiments with the three different models (RF, SVM, and ANN). We examined the cross-validation performance of the compounds encoded using the Morgan fingerprint and not the MACCS keys so we used the Morgan fingerprint with a radius of 3 and found similar results where, for RF, the mean was 0.83, the mean sensitivity was 0.84, the mean specificity was 0.81, and the mean AUC was 0.90. Furthermore, for SVM, the mean accuracy was 0.86, the mean sensitivity was 0.83, the mean specificity was 0.88, and the mean AUC was 0.90. Finally, for ANN, the mean accuracy was 0.86, the mean sensitivity was 0.85, the mean specificity was 0.87, and the mean AUC was 0.91.
2. Similarities search for Selumetinib: The random forest classifier was applied where the set model parameter for random forest estimators was 100, and the number of trees to grow criterion (entropy) and number cost function were optimized for a split. We observed that the sensitivity for RF was 0.88, the specificity for RF was 0.54, and the AUC for RF was 0.74. The support vector classifier was applied where the set model parameters for the SVM kernel were rbf, C value of 1, gamma value of 0.1, and the probability was True. We observed that the sensitivity for SVM was 0.94, the specificity was 0.46, and the AUC was 0.75. The neural network classifier was applied where the set model parameters for ANN hidden layer sizes were 5 and 3, and the random state was SEED. We observed that the sensitivity for ANN was 0.88, the specificity was 0.54 and the AUC was 0.75, as shown in Figure 9B; Table 3. We performed cross-validation experiments with the three different models (RF, SVM, and ANN). We examined the cross-validation performance of the compounds encoded using the Morgan fingerprint and not the MACCS keys so we used the Morgan fingerprint with a radius of 3 and found similar results where, for RF, the mean accuracy was 0.76, the mean sensitivity was 0.83, the mean specificity was 0.68 and the mean AUC was 0.83. Furthermore, for SVM, the mean accuracy was 0.78, the mean sensitivity was 0.88, the mean specificity was 0.64, and the mean AUC was 0.83. Finally, for ANN, the mean accuracy was 0.78, the mean sensitivity was 0.86, the mean specificity was 0.67, and the mean AUC was 0.83.
3. Similarities search for Lapatinib: The random forest classifier was applied where the set model parameter for random forest estimators was 100, and the number of trees to grow criterion (entropy) and number cost function were optimized for a split. We observed that the sensitivity for RF was 0.93, the specificity was 0.81, and the AUC was 0.92. The support vector classifier was applied where the set model parameters for the SVM kernel were rbf, C value of 1, gamma value of 0.1, and the probability was True. We observed that the sensitivity for SVM was 0.93, the specificity was 0.73, and the AUC was 0.90. The neural network classifier was applied where the set model parameters for the ANN hidden layer sizes were 5 and 3, and the random state was SEED. We observed that the sensitivity for ANN was 0.91, the specificity was 0.73, and the AUC was 0.89, as shown in Figure 9C; Table 3. We performed cross-validation experiments with all three different models (RF, SVM, and ANN). We examined the cross-validation performance of the compounds encoded using the Morgan fingerprint and not the MACCS keys so we used the Morgan fingerprint with a radius of 3 and found similar results where, for RF, the mean accuracy was 0.85, the mean sensitivity was 0.92, the mean specificity was 0.76, and the mean AUC was 0.92,. Furthermore, for SVM, the mean accuracy was 0.86, the mean sensitivity was 0.93, the mean specificity was 0.75, and the mean AUC was 0.91. Finally, for ANN, the mean accuracy was 0.82, the mean sensitivity was 0.89, the mean specificity was 0.73, and the mean AUC was 0.89.

**TABLE 3:** Performance of all three models (RF, SVM, and ANN).

Models	Accuracy (mean)	Sensitivity (mean)	Specificity (mean)	AUC (mean)	Accuracy (SD)	Sensitivity (SD)	Specificity (SD)	AUC (SD)
RF (Tanespimycin)	0.83	0.84	0.81	0.90	0.00	0.01	0.01	0.00
SVM (Tanespimycin)	0.86	0.83	0.88	0.90	0.00	0.02	0.02	0.01
ANN (Tanespimycin)	0.86	0.85	0.87	0.91	0.03	0.05	0.02	0.00
RF (Selumetinib)	0.76	0.83	0.68	0.83	0.08	0.01	0.18	0.06
SVM (Selumetinib)	0.78	0.88	0.64	0.83	0.07	0.07	0.20	0.07
ANN (Selumetinib)	0.78	0.86	0.67	0.83	0.06	0.04	0.20	0.04
RF (Lapatinib)	0.85	0.92	0.76	0.92	0.01	0.01	0.02	0.01
SVM (Lapatinib)	0.86	0.93	0.75	0.91	0.01	0.01	0.03	0.01
ANN (Lapatinib)	0.82	0.89	0.73	0.89	0.01	0.03	0.02	0.00

### Drugs-sensitive biomarkers identification

A biomarker is a naturally occurring entity with a specific pathological or physiological process that can be identified for therapeutic purposes. Here we inspected the association between the drugs and the screened biomarkers within two databases: CCLE and GDSC. We discovered that *SETD7*, a methyltransferase that catalyzes the monomethylation of lysine 4 on histone H3 is susceptible to all six medicines described in this study (Supplementary Figure S28) and a complete list of the drug-sensitive biomarkers is shown in Table 4. Several studies have revealed the role of *SETD7* in post-translational modifications of non-histone proteins. However, the predictive relevance of *SETD7* (Huang et al., 2017; Duan et al., 2018) in breast cancer and its ability to modulate intrinsic redox homeostasis has never been studied. *SETD7* aided tumor cell growth and inhibited apoptosis, as well as sensitively maintaining redox equilibrium by controlling GSH/GSSG and ROS levels.

**TABLE 4:** Extracting the drug sensitivity signatures from “rna” summarized experiment data.

ENSG00000145391	Lapatinib	Nilotinib	Panobinostat	PLX-4720	Sulemetinib	Tanespimycin
Estimate *p*-value	0.0443271136618988	0.0472448997625699	0.0412742045399219	0.0416579832978096	0.041129074405051	0.046394679732344
ENSG00000183888	Lapatinib	Nilotinib	Panobinostat	PLX-4720	Sulemetinib	Tanespimycin
Estimate *p*-value	−0.0188669660473501	0.0295980295254738	0.0587346546951706	0.035697820369925	0.0314155970023244	0.0341852186416538
ENSG00000145817	Lapatinib	Nilotinib	Panobinostat	PLX-4720	Sulemetinib	Tanespimycin
Estimate *p*-value	−0.0213867098786672	−0.223987174989867	0.045941414728702	0.0371605978309142	0.0326688348245422	0.0354858757422661

*SETD7* was found to be a positive activator of the KEAP1-NRF2 pathway in further research. *SETD7* is an antioxidant enzyme transcriptional activator. In MCF7 and MDA-MB cells, the downregulation of *SETD7* downregulates antioxidant enzymes and caused a redox imbalance. *SETD7* is a breast cancer prognostic marker and a new antioxidant promoter in the face of oxidative stress. Knockdown of *SETD7* inhibited cancer cell proliferation, induced G1/S cell cycle arrest, and increased apoptosis. Along with *SETD7*, we chose two other biomarkers known as *SRARP* and *YIPF5* (Suárez-Arroyo et al., 2016; Naderi, 2020) (Supplementary Figures S29, S30; Table 4). *SRARP*, which is found on chromosome 1p36, has recently been discovered as a new corepressor of the androgen receptor (AR). In breast cancer cell lines, primary breast tumors, and metastatic breast cancer, *SRARP* has been shown to be highly co-expressed with AR (Naderi, 2020). *SRARP* also has a fairly advanced countenance in breast tumors that are estrogen receptor-positive (ER+), lower grade, and lobular histology (Su et al., 2012; Naderi, 2018). Furthermore, functional investigations in breast cancer cells revealed an interaction between AR and *SRARP* (Naderi, 2018). Meanwhile, AR activation reduces *SRARP* transcription directly, and *SRARP*, in turn, engages with AR as a corepressor and inhibits AR-mediated production of prolactin-induced protein (PIP) and androgen response element reporter activity (Naderi, 2018). Furthermore, *SRARP*’s corepressor activity causes a decrease in AR binding to the PIP promoter (Naderi, 2018).

### Drugs and radiation-sensitive biomarkers identification

Radiogenomics is designed similarly to pharmacogenomics. The only difference is in the method of cell treatment. The only available clinical database which holds *in vitro* radiogenomics data is the Cleveland database. This dataset contains only gamma radiations. We used the SummarizeSensitivityProfiles function to retrieve radiation for a cell line summary of a sensitivity experiment. This yields a framework (matrix) with rows addressing the radiation type and columns addressing cell lines, representing values that sum up the viability measurements. Sensitivity measures can be specified using the sensitivity measure function.

The *YIPF5* (which stands for Yip1 domain family member 5) plays a role in transport between the endoplasmic reticulum and Golgi. *YIPF5* is a prognostic marker in head, neck, liver, and breast cancers.

*SETD7* was not observed to be sensitive to gamma radiation while the other two biomarkers (*SRARP* and *YIPF5*) were highly sensitive to the radiation (Table 5). The radiosensitive signatures *SRARP* and *YIPF5* are taken from the Cleveland database.

**TABLE 5:** Extracting the radiation sensitivity signatures (SRARP & YIPF5) from the Cleveland database.

Ensemble	Gene	Estimate	se	n	tstat	fstat	*p*-value	df	fdr
ENSG00000183888	SRARP	0.2589937	0.0510493	517	5.073401	25.73940	6.0e-07	493	0.0001125
ENSG00000145817	YIPF5	0.2521606	0.0474171	517	5.317924	28.28031	2.0e-07	493	0.0000497

In addition, we plotted the correlation coefficient of the shortlisted biomarkers with all of the proposed drugs, with the Pearson correlation coefficient threshold set at | 0.7|. Drug correlation coefficients greater than the threshold were considered to have a strong correlation with biomarkers. The correlation coefficients for *SETD7, SRARP*, and *YIPF5* are given in Supplementary Figures S6–S8 respectively. A positive correlation suggested that cells responding to Lapatinib, Selumetinib, and Tanespimycin treatment differ from those responding to radiation. The negative correlation between the radiation response signature and drug response (Nilotinib, Panobinostat, and PLX4720) suggests that these drugs could be used as a radiosensitizing agent in conjunction with ionizing radiation to improve treatment efficacy. The signatures’ negative correlation can be interpreted to predict that radiation and drugs (Nilotinib, Panobinostat, and PLX4720) would target different cell populations in a tumor. The radiation score and *p*-values of all the shortlisted drugs are given in Table 6.

**TABLE 6:** Comparing sensitivity signatures between radiation and drug response.

Drugs	Radiation score	*p*-value
Lapatinib	0.579293195	0.009950249
Nilotinib	−0.324758293	0.009950249
Panobinostat	−0.491200093	0.009950249
PLX4720	−0.196630872	0.009950249
Selumetinib	0.571017489	0.009950249
Tanespimycin	0.293541670	0.009950249

### Drugs and radiation-resistance biomarkers identification

Radioresistance always has been a key roadblock in the advancement of radiation treatment. The contents of liberated extracellular vesicles vary as a result of radiotherapy. Exosomes generated from irradiation cells have been demonstrated to impact host cell proliferation, motility, cell cycle arrest, and death, according to studies. Exosomes appear to have a key role in radioresistance, according to the data. The radioresistant signatures from the Cleveland database were also extracted but no radioresistant signatures were found for breast cancer (Table 7).

**TABLE 7:** Listing the radio-resistant signatures data from Cleveland database. There is no radio-resistant biomarker being observed for breast cancer.

Ensemble	Gene	Estimate	se	n	tstat	fstat	*p*-value	df	fdr
ENSG00000189007	ADAT2	−0.2668198	0.0473300	517	−5.637440	31.78073	0	493	2.23e-05
ENSG00000004487	KDM1A	−0.2681836	0.0436481	517	−6.144227	37.75152	0	493	3.80e-06
ENSG00000160208	RRP1B	−0.2715109	0.0434827	517	−6.244118	38.98902	0	493	3.80e-06
ENSG00000113356	POLR3G	−0.2785138	0.0454217	517	−6.131739	37.59823	0	493	3.80e-06
ENSG00000148835	TAF5	−0.2852464	0.0433393	517	−6.581702	43.31880	0	493	7.00e-07
ENSG00000129351	ILF3	−0.2929693	0.0426313	517	−6.872171	47.22673	0	493	3.00e-07

## Conclusion

Chemotherapy is the most frequent systemic treatment for triple-negative breast cancer (TNBC) patients in the early stages as well in the late stages of the progression of the disease. TNBC patients have a poor prognosis, as a result, a considerable effort has been made so that we can find responsive molecular targets to treat these malignancies. Although the accessibility of data has been increased due to the high throughput sensitivity of drug testing, effective drug response still remains a challenge. Understanding the interaction between a cell line and a specific drug will eventually allow for tailored treatment for specific cancer patients (Zhao et al., 2015). These results demonstrate the transcriptional effects of derivatives (screened against approved drugs) across a pool of cell lines and highlight the utility of such information for identifying a drug’s cellular effects and mechanism of action.

In this study, we predicted drug sensitivity on breast cancer cell lines, out of which three main biomarkers were shortlisted by evaluating their response to the drugs and exposure to radiation. In our case of predicting drug sensitivity, the highest accuracy was found for PLX-4720 drugs using a random forest approach. Three main biomarkers, *SETD7, SRARP,* and *YIPF5,* were identified. *SETD7* was not radiosensitive, while *SRARP* and *YIPF5* showed sensitivity to all the drugs and gamma radiations from the Cleveland database. Additionally, no radioresistant biomarkers were found for TNBC. The main limitation was the accuracy limit, which is insignificant because of the low availability of data. Accuracy can be further improved when more data become available. Here we are specifically focusing on the TNBC data only. In future studies, we can improve model performance by considering more data and including single-cell data for drug and biomarker screening.

In our study, we attempted to provide a solid groundwork for machine learning-driven prediction of drug sensitivity for TNBC, which has not been reported previously at this level, and the shortlisted markers could be potential therapeutic targets. Future research will likely focus on computational and experimental molecular modeling of shortlisted drugs and biomarkers. This understanding will bring the era of personalized cancer medicine closer to reality.

## Data Availability

The datasets presented in this study can be found in online repositories. The names of the repository/repositories can be found in the article/Supplementary Material. All relevant data are within the paper and its Supporting Information files. The cancer cell line data were manually downloaded from Cancer Cell Line Encyclopedia (CCLE) (https://sites.broadinstitute.org/ccle/), and cell lines and drug profile data were manually downloaded from Genomics of Drug Sensitivity in Cancer (GDSC) (https://www.cancerrxgene.org). For script and CCLE1.0, GDSC2.0 and Cleveland1.0 data and their links can be found in GitHub repositories https://github.com/ML-PDDT/TNBC/tree/main.
