# Supplementary material for: Machine learning-driven exploration of drug therapies for triple-negative breast cancer treatment
Source: Front Mol Biosci. 2023 Aug 4;10:1215204. doi: 10.3389/fmolb.2023.1215204 (PMC10436744; doi:10.3389/fmolb.2023.1215204)
Supplement: Supplementary file 1 [file DataSheet1.docx]

**Machine Learning-Driven Exploration of Drug Therapies for Triple Negative Breast Cancer Treatment**





**Figure S1.** The area above the curve (AAC) for all six drugs on various breast cancer cell lines. The red color represents CCLE while the blue one stands for the GDSC database.


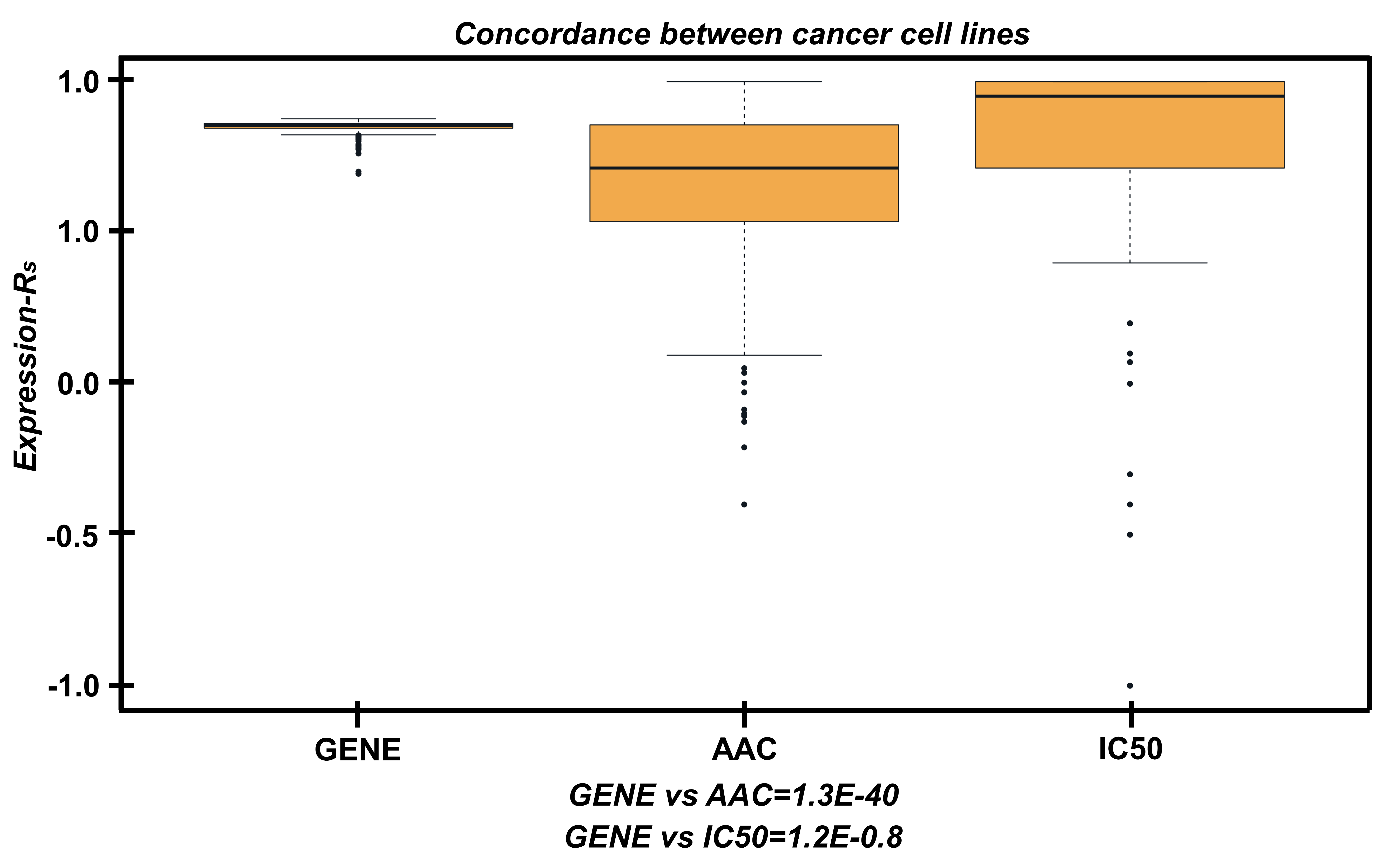


**Figure S2.** Summarizing the gene, area above the curve (AAC), and IC50 details of the drugs on breast cancer cell lines.





**Figure S3.** The IC50 of all the six drugs on various breast cancer cell lines. Not all the drugs are mentioned because very few experiments have been conducted using these drugs. The red color represents CCLE while the blue one stands for the GDSC database.

**
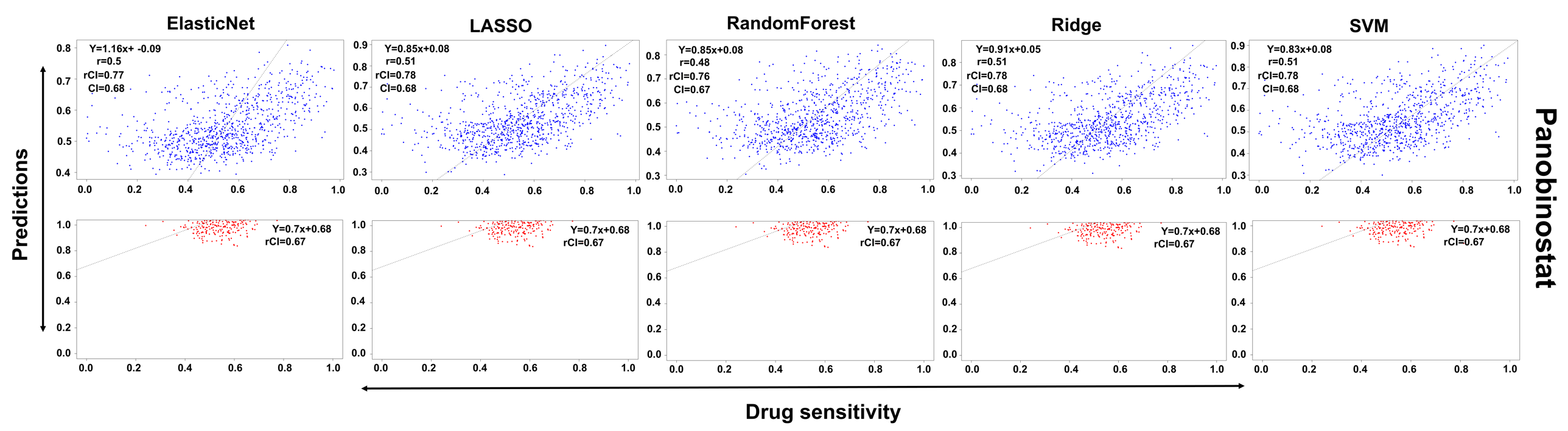
**

**Figure S4.** Predictions and validations using five machine learning methods for Panobinostat. The blue dots represent predictions while red refers to the validations.


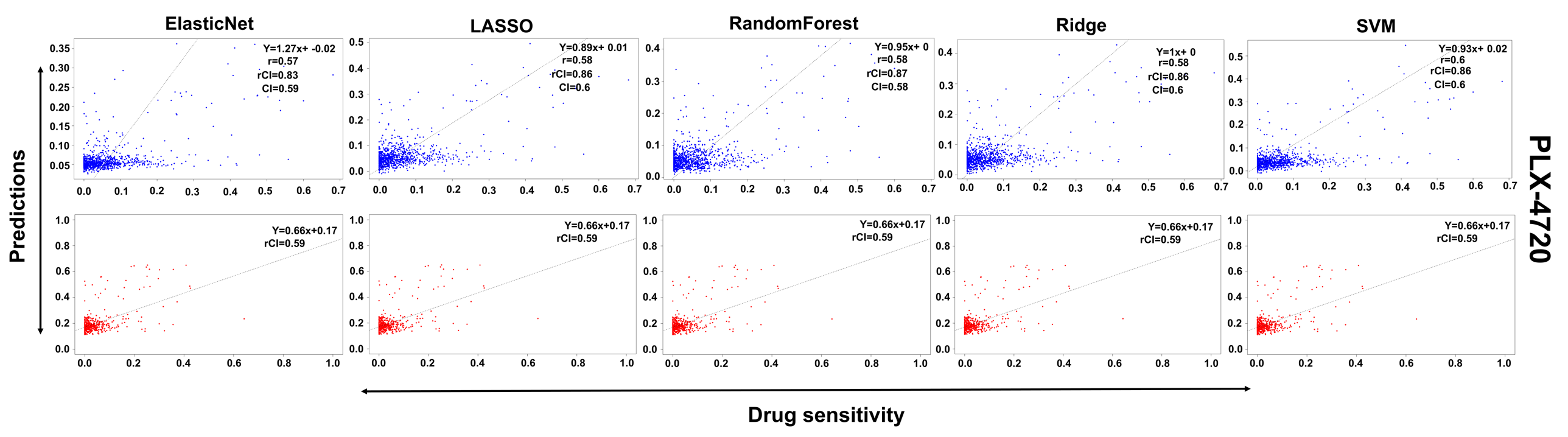


**Figure S5.** Predictions and validations using five machine learning methods for PLX-4720. The blue dots represent predictions while red refers to the validations.

**Data and methods**

**Similarity Search using Machine learning**

**Data retrieval for shortlisted targets:** We took heat shock protein 90 (HSP90) for Tanespimycin, Mitogen-activated protein kinase (MEK) for Selumetinib and Human epidermal growth factor receptor 2 (ErbB2) for Lapatinib for further evaluation. Bioactivity data for all targets were retrieved as pChEMBL (pChEMBL prediction of a small molecule over protein targets, are based on the QSAR and Machine Learning models built on RDK fingerprints and activity dataset available from ChEMBL database) values. The ligand-based virtual screening analysis was performed using our own written script in Python against the ChEMBL library of compounds.

**Pre-processing of the bioactivity compounds:** We analyzed the data from the ChEMBL library of compounds to filter bioactivity compounds for ovarian cancer. First, we converted the standard value datatype from "object" to "float" and then removed entries with missing molecule structure entries. We kept only entries with standard units (nM) and removed duplicate molecules. Molecules with canonical SMILES were retained while all molecules without canonical SMILES were removed before further evaluation. Finally, we converted IC50 to pIC50 (log10(IC50)).

**Labeling of active compounds with the rule of five (Lipinski)**: We labeled those compounds having values of less than 1000 nM (considered to be active) while those greater than 10,000 nM (considered to be inactive) as for those values in between 1,000 and 10,000 nM (referred to as intermediate) for further evaluations. Then we did Lipinski calculation for evaluating the drug-likeness of compounds. Drug likeness is based on the pharmacokinetic profile which is represented by Absorption, Distribution, Metabolism and Excretion (ADME). Lipinski analyzed all orally active FDA-approved drugs to establish the Rule of Five, which states the following: 1) molecular weight should be < 500 Dalton, 2) octanol-water partition coefficient (LogP) should be < 5, 3) hydrogen bond donors should be < 5 and 4) hydrogen bond acceptors < 10. 5) cLogP < 5

**Calculate fingerprint descriptors**: We used the MACCS and Morgan fingerprints for the Tanespimycin, Selumetinib, and Lapatinib molecules, and then generated MACCS and Morgan fingerprints for all molecules in the processed dataset. Calculated the Tanimoto similarity (**substructure searching**) between the query molecule (Tanespimycin) and all molecules in our processed dataset (using MACCS and Morgan fingerprints).

1. **Tanespimycin:** 408 compounds converted where fingerprint length per compound was 2048 and Tanimoto similarity (0.25) and distance matrix (0.75) while the calculated distance matrix (distance = 1-similarity) was observed to be 0.7481371087928466, 0.8108419838523645, 0.7469437652811737, 0.8123359580052494, and 0.7320169252468265. We also calculated the number of elements in the triangular matrix via n*(n-1)/2 and found that the Tanimoto distance matrix (fingerprints) was 83028.

**Clustering of the molecules based on their fingerprint similarity for Tanespimycin:** Ran the clustering procedure for the entire dataset and found the total number of clusters was 68 where the number of clusters with only 1 compound was39, the number of clusters with >5 compounds was 14, number of clusters with >25 compounds was 5 and number of clusters with >100 compounds was 0.

**Similarities search for Tanespimycin using machine learning**: First, we did data preparation or data labeling where we added a column for activity with a pIC50 of >= 6.0, and we found the number of active compounds was 210 while the number of inactive compounds was 198. Molecule encoding was done using the MACCS Method and we applied three Classical Machine learning approaches to classify our molecules named Random Forest (RF), Support Vector Machine (SVM), and Artificial Neural Network (ANN) and performed performance of models where we fit classical machine learning models on a train-test split of the data. Splitting the data was reused for the two other classical models, we used test (x) and train (x) for the respective fingerprint splitting and test (y) and train (y) for the respective label splits, where training data size was 326 and test data size was 82.

1. **Selumetinib:** 144 compounds converted where fingerprint length per compound was 2048 and Tanimoto similarity (0.19) and distance matrix (0.81) while calculated distance matrix (distance = 1-similarity) was observed to 0.8132295719844358, 0.699288256227758, 0.8260869565217391, 0.6932038834951456, and 0.8606060606060606. We also calculated the number of elements in the triangular matrix via n*(n-1)/2 and found that the Tanimoto distance matrix (fingerprints) was 10296.

**Clustering of the molecules based on their fingerprint similarity for Selumetinib:** Ran the clustering procedure for the entire dataset and found the total number of clusters was 48 where the number of clusters with only 1 compound was32, the number of clusters with >5 compounds was 7, number of clusters with >25 compounds was 0, and number of clusters with >100 compounds was 0.

**Similarities search for Selumetinib using machine learning**: First, we did data preparation or data labeling where we added a column for activity with a pIC50 of >= 6.0, and we found the number of active compounds was 93 while the number of inactive compounds was 51. Molecule encoding was done using MACCS Method and we applied three Classical Machine learning approaches to classify our molecules named Random Forest (RF), Support Vector Machine (SVM), and Artificial Neural Network (ANN) and performed performance of models where we fit classical machine learning models on a train-test split of the data. Splitting the data was reused for the two other classical models, we used test (x) and train (x) for the respective fingerprint splitting and test (y) and train (y) for the respective label splits, where training data size was 115 and test data size was 29.

1. **Lapatinib:** 1277 compounds converted where the fingerprint length per compound was 2048 and Tanimoto similarity (0.93) and distance matrix (0.07) while the calculated distance matrix (distance = 1-similarity) was observed 0.07235142118863047, 0.14035087719298245, 0.2009569377990431, and 0.2009569377990431, 0.13691931540342295. We also calculated the number of elements in the triangular matrix via n*(n-1)/2 and found that the Tanimoto distance matrix (fingerprints) was 814726.

**Clustering of the molecules based on their fingerprint similarity for Lapatinib:** Ran the clustering procedure for the entire dataset and found the total number of clusters was 229 where the number of clusters with only 1 compound was116, the number of clusters with >5 compounds was 56, number of clusters with >25 compounds was 8, and number of clusters with >100 compounds was 0.

**Similarities search for Lapatinib using machine learning**: First, we did data preparation or data labeling where we added a column for activity with a pIC50 of >= 6.0, and we found the number of active compounds was 735 while the number of inactive compounds was 542. Molecule encoding was done using the MACCS Method and we applied three Classical Machine learning approaches to classify our molecules named Random Forest (RF), Support Vector Machine (SVM), and Artificial Neural Network (ANN) and performed performance of models where we fit classical machine learning models on a train-test split of the data. Splitting the data was reused for the two other classical models, we used test (x) and train (x) for the respective fingerprint splitting and test (y) and train (y) for the respective label splits, where training data size was1021 and test data size was 256.

**Results**

**Figure S6:** Radar plot for the dataset of compounds that fulfill the Rule of five where **a.** depicts Tanespimycin **b.** depicts Selumetinib and **c.** depicts Lapatinib.

**Figure S7:** Distribution of similarity values for the query molecule (**Tanespimycin**) and all the molecules using MACCS and Morgan.

**Figure S8:** Comparative analysis between Tanimoto and Dice similarities for the two fingerprints for Tanespimycin.

**Figure S9:** **Plot of the clusters:** clustering of the molecules based on their fingerprint similarity for Tanespimycin.


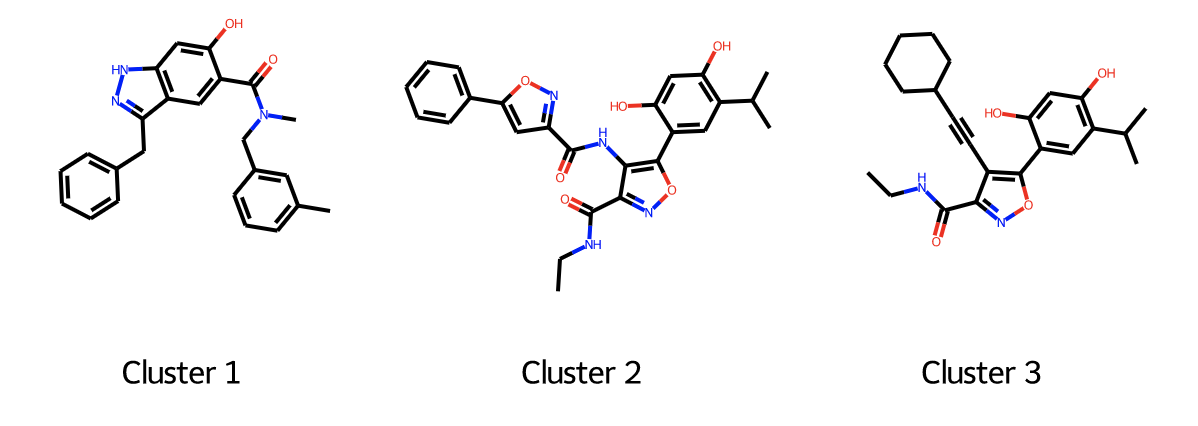


**Figure S10:** Plot of first 3 clusters where **the** number of molecules in the largest cluster was similar to between two random points in the same cluster of Tanespimycin.

**Figure S11:** Similarity between two fingerprints or clusters using Tanimoto similarities where similarity metrics were compared based on their ranking of the compounds for Tanespimycin.


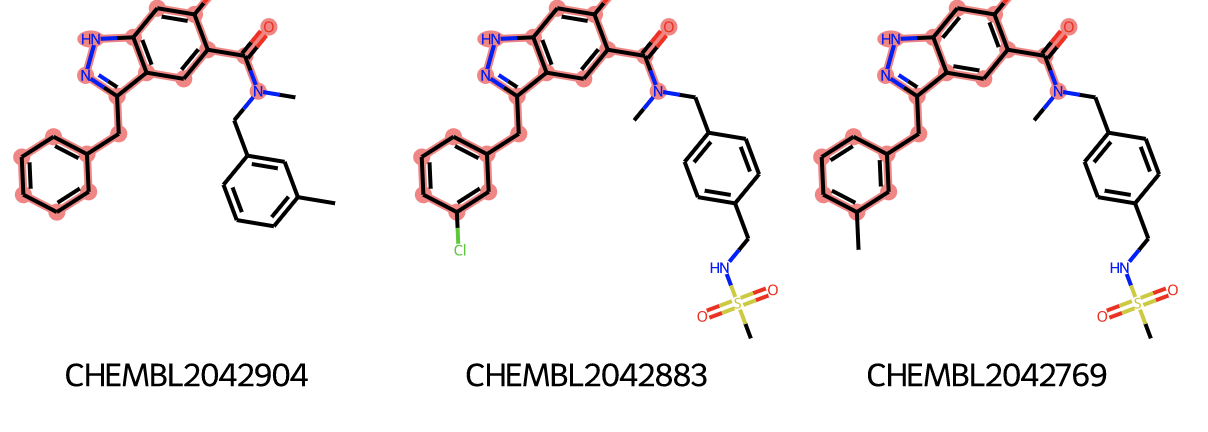
**Figure S12:** Calculation of maximum common substructure using FMCS algorithm for Tanespimycin.


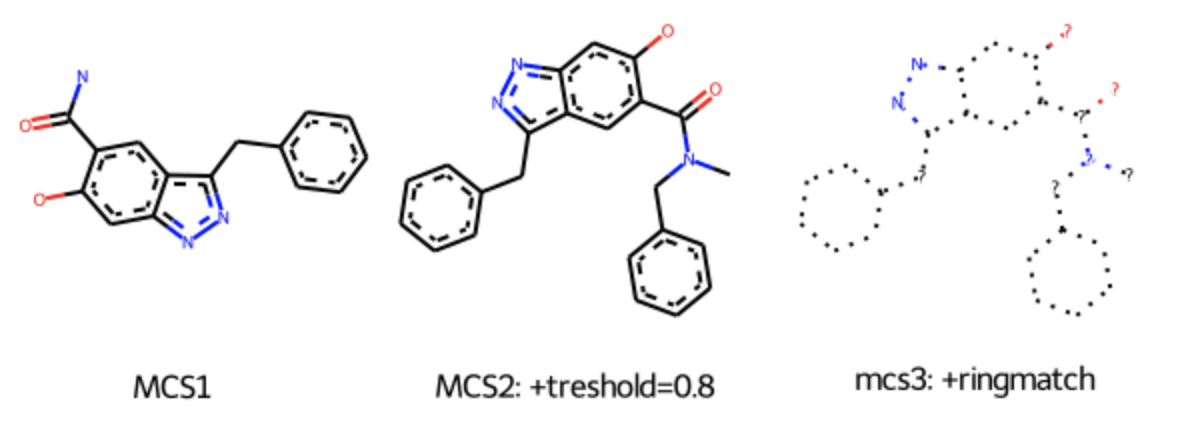


**Figure S13: Substructure:** matched ring bonds where MCS2 contains 28 atoms and 31 bonds while MCS3 contains 28 atoms and 31 bonds for Tanespimycin.

**Figure S14:** Distribution of similarity values for the query molecule (**Selumetinib**) and all the molecules using MACCS and Morgan.

**Figure S15:** Comparative analysis between Tanimoto and Dice similarities for the two fingerprints for Selumetinib.

**Figure S16:** **Plot of the clusters:** clustering of the molecules based on their fingerprint similarity for Selumetinib.


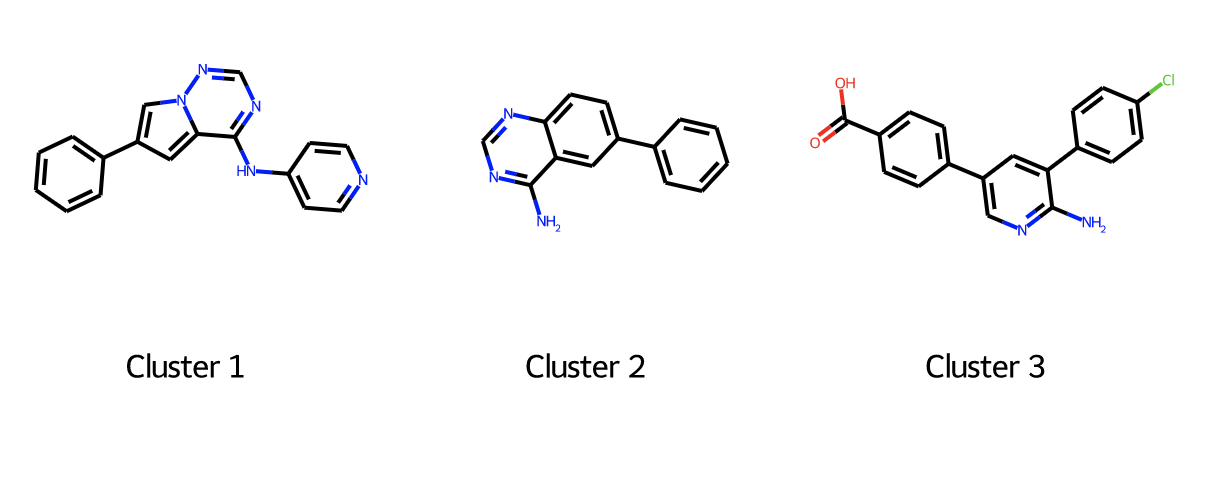


**Figure S17:** Plot of first 3 clusters where **the** number of molecules in the largest cluster was similar to between two random points in the same cluster of Selumetinib.

**Figure S18:** Similarity between two fingerprints or clusters using Tanimoto similarities where similarity metrics were compared based on their ranking of the compounds for Selumetinib.


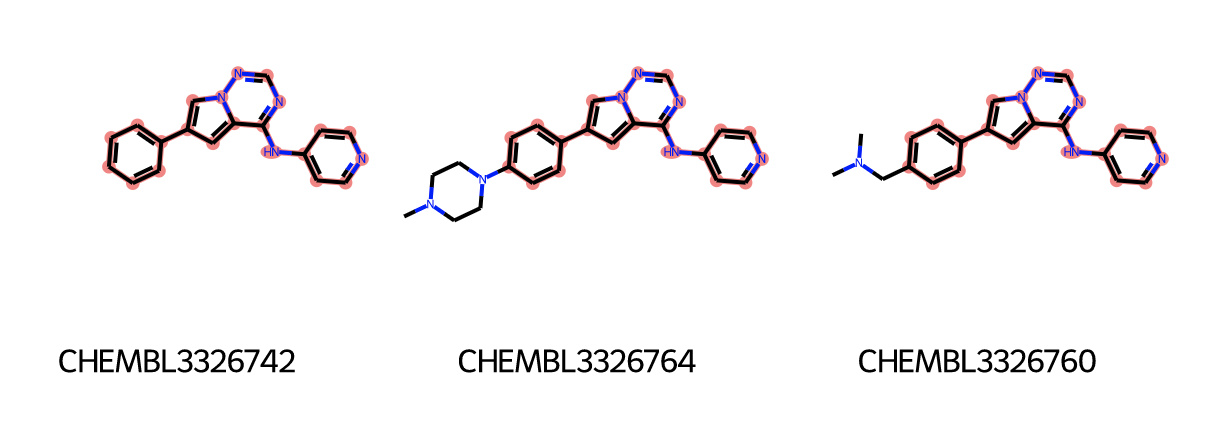


**Figure S19:** Calculation of maximum common substructure using FMCS algorithm for Selumetinib.


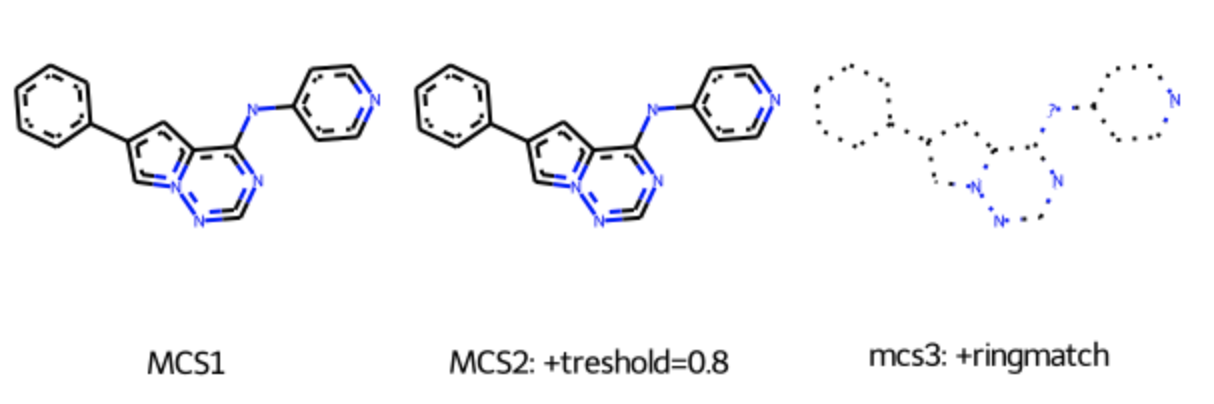


**Figure S20: Substructure:** matched ring bonds where MCS2 contains 22 atoms and 25 bonds while MCS3 contains 22 atoms and 25 bonds for Selumetinib.

**Figure S21:** Distribution of similarity values for query molecule (**Lapatinib**) and all molecules using MACCS and Morgan.

**Figure S22:** Comparative analysis between Tanimoto and Dice similarities for the two fingerprints for Lapatinib.

**Figure S23:** **Plot of the clusters:** clustering of the molecules based on their fingerprint similarity for Lapatinib.


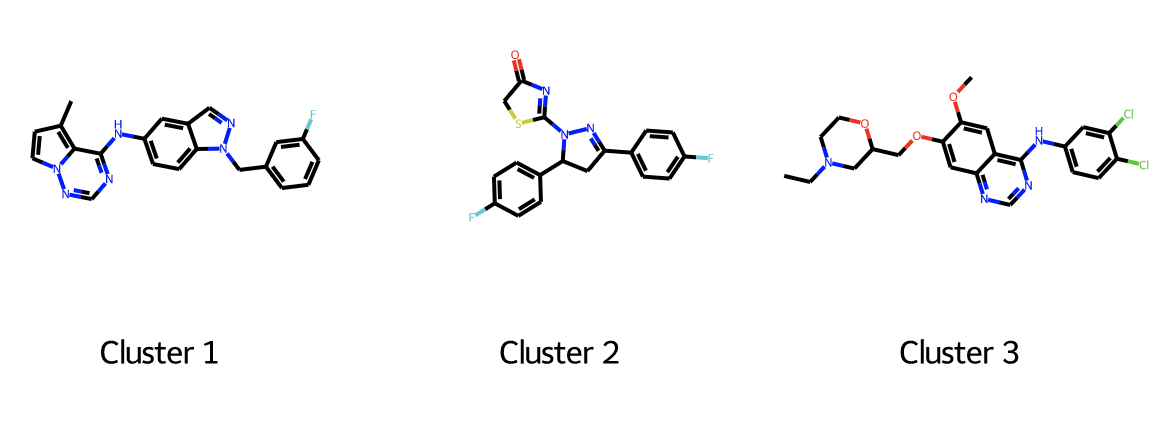


**Figure S24:** Plot of first 3 clusters where **the** number of molecules in the largest cluster was similar to between two random points in the same cluster of Lapatinib.

**Figure S25:** Similarity between two fingerprints or clusters using tanimoto similarities where similarity metrics were compared based on their ranking of the compounds for Lapatinib.


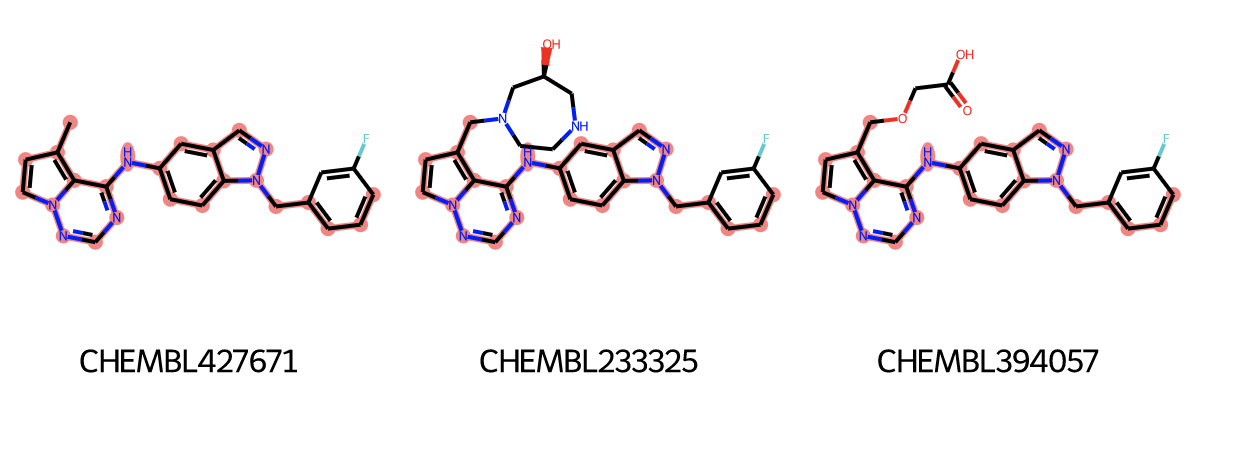


**Figure S26:** Calculation of maximum common substructure using FMCS algorithm for Lapatinib.


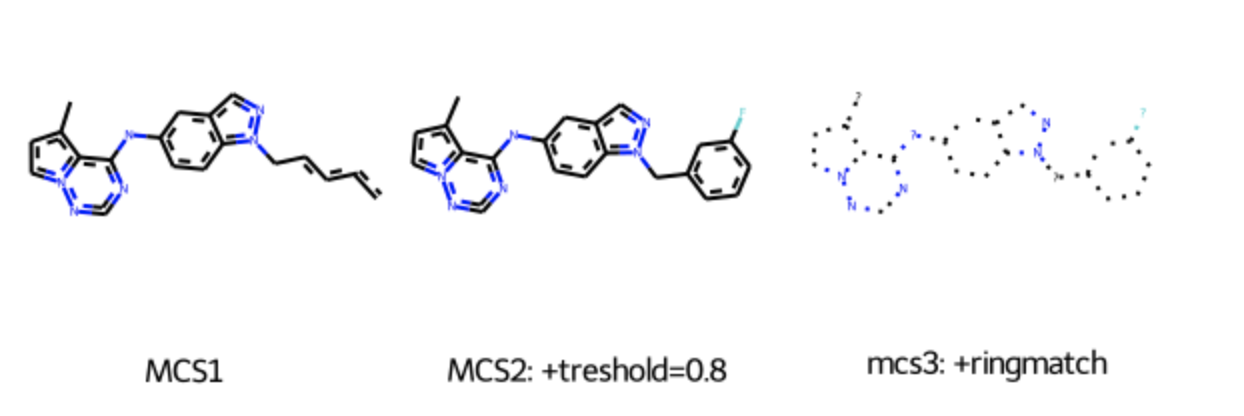


**Figure S27: Substructure:** matched ring bonds where MCS2 contains 28 atoms and 32 bonds while MCS3 contains 28 atoms and 32 bonds for Lapatinib.


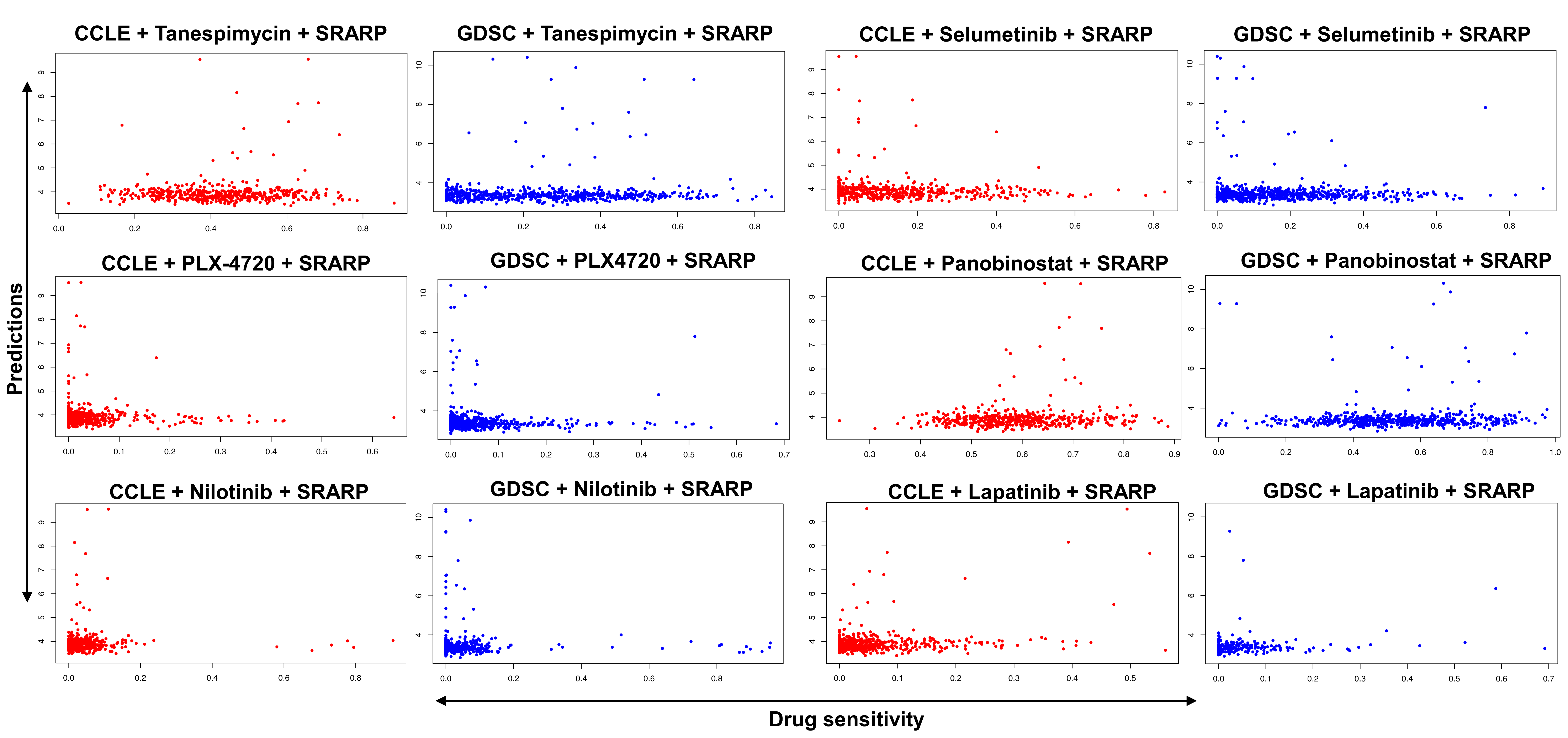

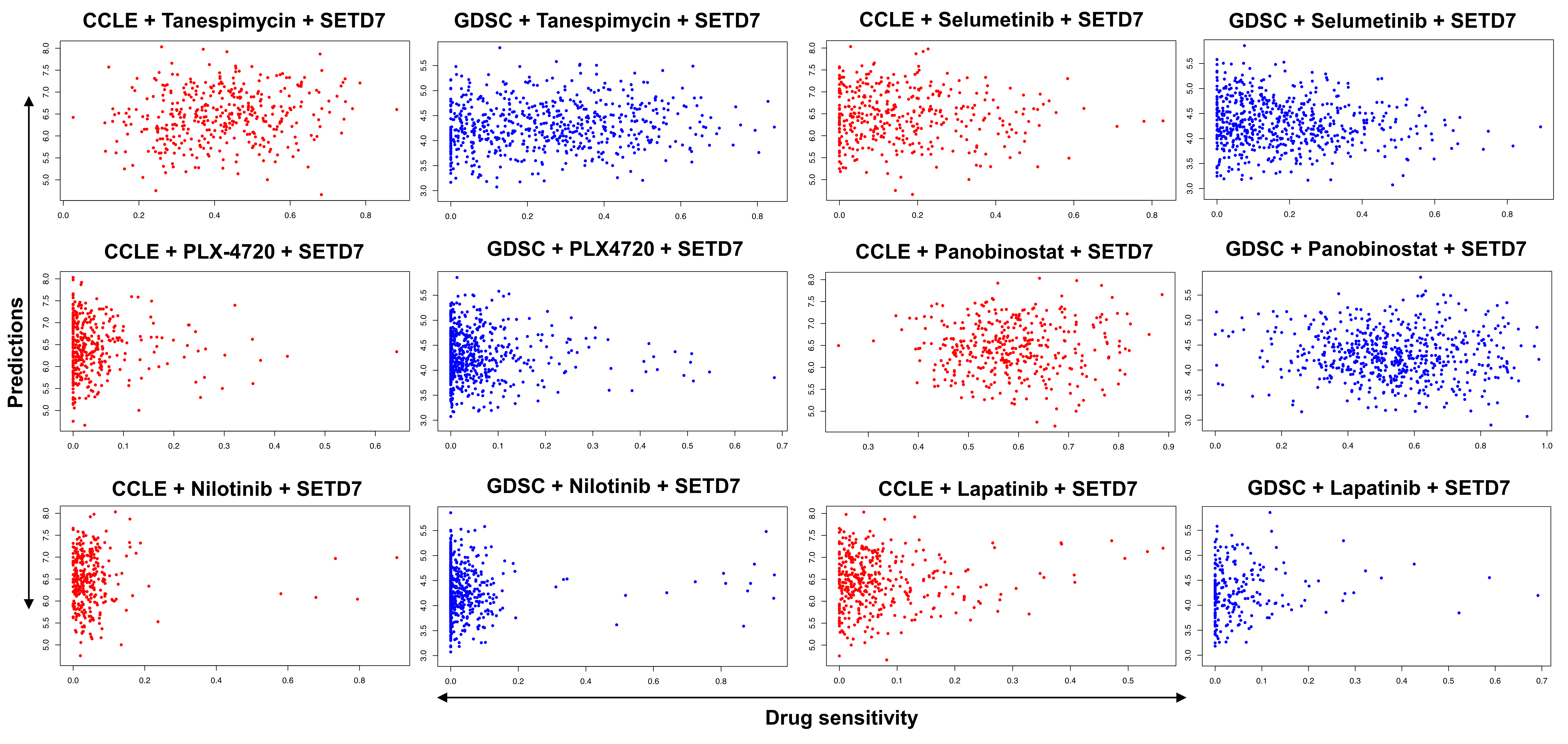
**Figure S28.** Breast tissue biomarker (SETD7) having a P-value of 0.0010970. Here the prediction is made from the combination of drug, SETD7 biomarker, and cell lines data.


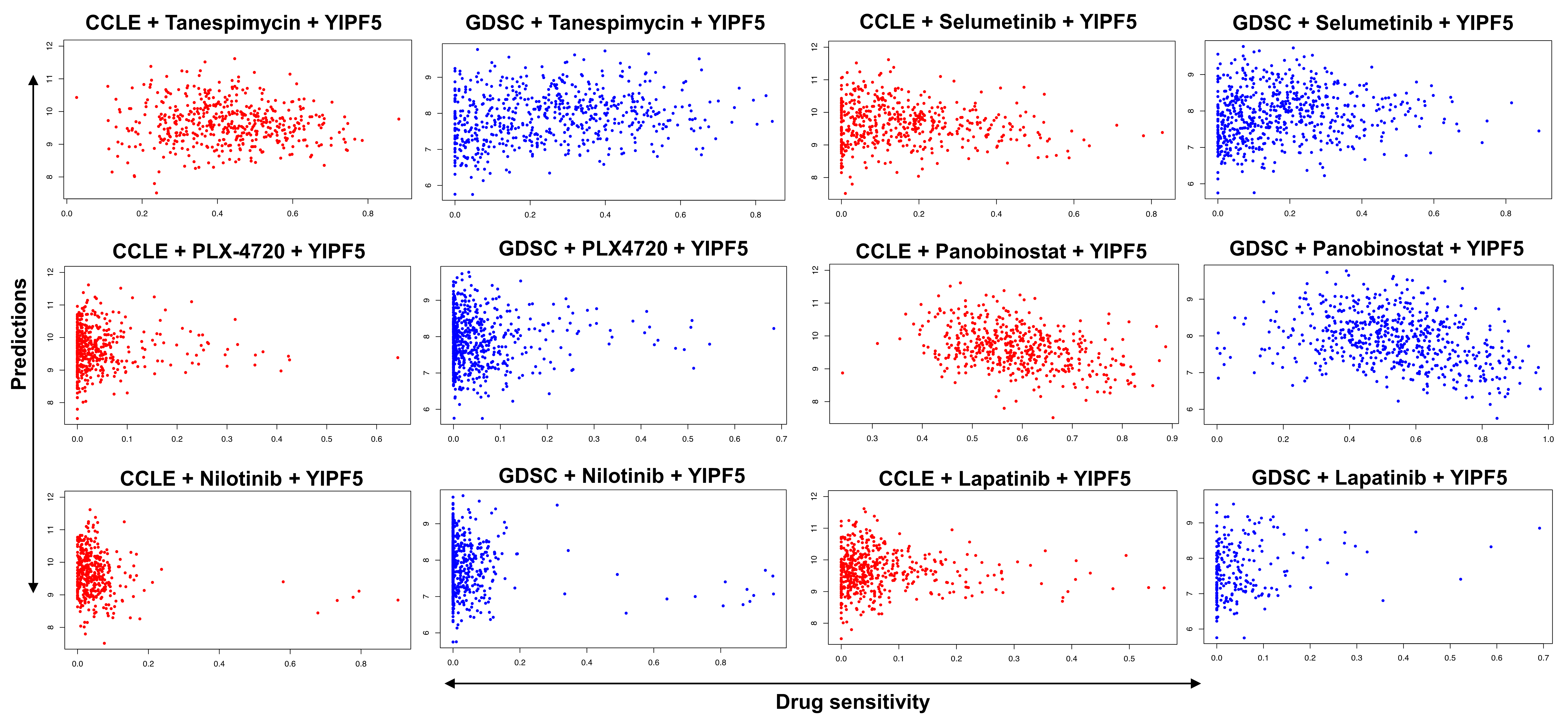
**Figure S29.** Radiation sensitivity predictions for SRARP on breast cancer cell lines. The P-value is observed to be 6.0e-07.

**Figure S30.** Radiation sensitivity predictions for YIPF5 on breast cancer cell lines. The observed P-value is 2.0e-07.

*******
